# Synchronous facial action binds dynamic facial features

**DOI:** 10.1038/s41598-021-86725-x

**Published:** 2021-03-30

**Authors:** Alan Johnston, Ben B. Brown, Ryan Elson

**Affiliations:** School of Psychology, University Park, The University of Nottingham, Nottingham, NG7 2RD UK

**Keywords:** Psychology, Motion detection, Object vision

## Abstract

We asked how dynamic facial features are perceptually grouped. To address this question, we varied the timing of mouth movements relative to eyebrow movements, while measuring the detectability of a small temporal misalignment between a pair of oscillating eyebrows—an eyebrow wave. We found eyebrow wave detection performance was worse for synchronous movements of the eyebrows and mouth. Subsequently, we found this effect was specific to stimuli presented to the right visual field, implicating the involvement of left lateralised visual speech areas. Adaptation has been used as a tool in low-level vision to establish the presence of separable visual channels. Adaptation to moving eyebrows and mouths with various relative timings reduced eyebrow wave detection but only when the adapting mouth and eyebrows moved asynchronously. Inverting the face led to a greater reduction in detection after adaptation particularly for asynchronous facial motion at test. We conclude that synchronous motion binds dynamic facial features whereas asynchronous motion releases them, allowing adaptation to impair eyebrow wave detection.

## Introduction

Facial movement supports interpersonal communication and familiar facial gestures can provide clues to identity^1–4^. However, it is unlikely that we encode every possible dynamic configuration of the face as a sequence of images due to the high requirements for information storage this implies. Instead, the perceptual system might take advantage of the regularities in facial action to encode dynamic information in a reduced form^5–9^. But what is the precise nature of this representation in human vision?

Although systems for describing facial actions, such as FACS, are well established^10^, it remains to be determined how dynamic features are grouped in the perceptual system and the brain. We sought to discover whether there are high-level dynamic configurations of the face that would be subject to adaptation. Adaptation can change appearance or alter sensitivity to visual information. Arguably, adaptation that raises detection threshold provides stronger evidence for the presence of tuned mechanisms than a shift in a category boundary, such as one finds in adaptation to facial appearance^11,12^. We therefore focus here on the effects of adaptation on perceptual performance rather than facial appearance.

The facial form is constrained by the facial skeleton and musculature^13^. The configuration of the lower half of the face is largely controlled by the movement of the jaw and the upper part of the face is largely controlled by the muscles around the eyes such as the occipitofrontalis, which raises the eyebrow and wrinkles the forehead. The upper and lower parts of the face are free to move independently, however, evidence is accruing that dynamic facial features are not processed in isolation.

Like walking^14^, facial action, and facial speech in particular, is approximately harmonic; features move away from their neutral position and later return. The presence of sinusoidal mouth movement can slow the apparent speed of eyelid closure, but only for feature motion phases in which the eyes close while the mouth opens or in which mouth opening leads eye opening^15^. This implies that the eyelid motion can become bound to the mouth movement resulting in a perceptual slowing of the eyelid closure. A more direct measure of binding utilises the observation that there is a generic upper limit for reporting feature pairings in two alternating feature sequences of around 3 Hz^16^, which can be exceeded if the feature pairs are perceptually coded as conjunctions^17,18^. Harrison, et al.^19^ showed that the conjunction of eye gaze direction and eyebrow position can be reported at high alternation rates (approx. 8 Hz) demonstrating feature binding, but the conjunction of eye gaze direction and mouth opening could only be reported at the generic rate of around 3–4 Hz, suggesting that lateral eye movements and vertical jaw movements may be processed independently. The Harrison et al. study chose to pair lateral movements of the eyes with vertical movements of the mouth as Maruya et al.^20^ had showed that temporal limits for reporting motion direction combinations for spatially separated low-level motion stimuli were much higher (approx. 10 Hz) for same and opposite motion directions than for horizontal and vertical directions (approx. 3 Hz) in a “T” configuration. This observation constrains the kinds of facial feature movement that can investigated with this technique. Taken as a whole, these studies provide evidence that only some features are perceptually bound, that relative timing is critical, and that perceptual grouping in faces mirrors coordinated action^8^. In this study we chose to use eyebrow and jaw movement, which together control correlated global change in the face, as eye gaze direction and eye opening and closing can be relatively independent of global changes in face shape.

Given that coordinated action of the eyebrows and jaw control much of the global configuration of the face, we considered two options for global dynamic feature coding, one based on a population code for relative motion and the other based on a rate code. On a population code, we would expect expressions to be encoded by matching to discrete channels, each coding for a specific brow and mouth temporal offset or phase shift. On a rate code, information is coded as a magnitude along some set of dimensions. Recent work has established that face identity is coded in this way^21^. For dynamic cyclical change in the face, one would naturally wish to code configuration as a phase, in our case expressing the relative motion of the mouth and eyebrows. This could be extracted explicitly as the arctangent of the ratio of activations in just two broad channels, encoding synchronous and asynchronous motion respectively. The synchronous channel would be most active when the eyebrows and jaw moved up and down together. The asynchronous channel would be most active when the eyebrows and jaw moved with an intermediate temporal offset. Alternatively, the information could be coded implicitly as a pair of rates, indicating the proportion of synchronous and asynchronous motion respectively, just as the position of a point on a circle is coded by a pair of sine and cosine functions. Other schemes may be possible, but these considerations guided the design of our first experiment.

If configural change in the face was encoded in discrete channels, we would only expect to see an effect of adaptation when the test stimulus matched the adaptor. If dynamic change was encoded in terms of two broad synchronous and asynchronous channels we would expect adaptation to be greater when the adaptor and test matched in terms of their synchronicity. In each condition we adapted participants to oscillating eyebrow motion and tested the effects of adaptation on the detection of an eyebrow wave, generated by a small temporal offset between oscillating eyebrows, in one of two test faces. To target systems encoding the relative motion of facial features, we varied the timing of mouth movement relative to the eyebrow movement at both adaptation and test. We found that rather than adaptation being specific to matching adapt and test phases, adaptation was only seen in the case of asynchronous adaptors and it affected all test phases equally.

## Experiment 1

### Method

#### Participants

Fifteen healthy adults (8 male) participated in Experiment 1. Sample size was decided a priori. All participants provided written, informed consent. The study was approved by the Ethics Committees of University College London and the University of Nottingham. All procedures adhered to the guidelines of the Declaration of Helsinki, 2008. Participants were screened for corrected visual acuity (20/20 or above) using a Snellen Chart before participating.

#### Design and procedure

**Figure 1:**
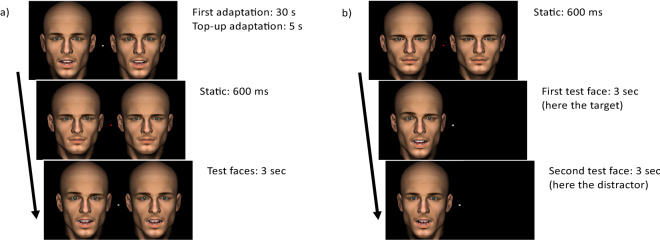
Stimulus and timeline. **(a)** Experiment 1: After adaptation to eyebrow and mouth relative movement the face was static for 600 ms followed by a 3 s period during which the dynamic test images were displayed either side of fixation. The eyebrows and jaw movements were sinusoidal (1.5 Hz) and the relative phase of mouth movements relative to eyebrow movements were either 0° (mouth opening; eyebrows dropping), 90° (mouth opening after eyebrows rising), 180° (mouth opening; eyebrows rising) and 270° (mouth opening after eyebrows dropping). All four phases were used at both adaptation and test providing 16 adaptation conditions. Four additional no-adaptation control conditions made for a total of 20 conditions. One of each pair of test faces contained misaligned eyebrow movements. The misalignment consisted of a roughly at-threshold temporal phase offset between the eyebrows of the target. The distractor face eyebrows were always aligned. Both test faces contained the same eyebrow-mouth relative timing in each trial. **(b)** Experiment 3: All dynamic test faces were presented to either the left or right visual fields. There was no adaptation period. Each trial was preceded by a 600 ms interval containing a static face. The two 3 s test intervals were distinguished by an abrupt change in phase. Eyebrow misalignment detection was measured at all 4 phases. Faces were also presented upright and inverted. The stimuli were generated using Poser Pro 9 (SmithMicro; https://www.posersoftware.com).

Participants performed a two-alternative-forced-choice discrimination task after adaptation to facial movement (Fig. 1a). Each block began with 30 s of initial adaptation and each trial was preceded by 5 s of top-up adaptation. At the adaptation stage a pair of identical moving faces appeared simultaneously to the left and right of fixation. At the test stage the adaptors were replaced by two faces presented for 3 s each, followed by an interval of at least 1 s during which the participants could make their response. The participant’s task was to report, by a keypress, which test face contained a temporal misalignment of 12.5° of phase in its eyebrows. The misalignment straddled the two eyebrows such that from onset of the eyebrow pair one eyebrow led by 6.25° whereas the other trailed by 6.25°. The eyebrows were at their low point at onset and which eyebrow led was randomised over trials. The 12.5° phase shift was chosen to deliver a level of performance of around 75% correct.

The stimuli were generated using Poser Pro 9 (SmithMicro; https://www.posersoftware.com). The mouth movement resulted from a lowering of the jaw. Both the eyebrow movement and the jaw movement were sinusoidal changes of position. The frequency of the motion was 1.5 Hz. This rate was chosen to provide a pace of movement that was consistent with natural facial action. In the adaptation phase the mouth motion was offset relative to the eyebrows by 0°, 90°, 180° or 270° of phase. For the test phase the mouth oscillations were again offset relative to the eyebrows by 0°, 90°, 180° or 270°. Zero phase was defined as upward movement of both the chin and eyebrows. A phase of 180° describes upward movement of the eyebrow combined with the downward movement of the chin, as in expressing surprise. At 90° of phase the mouth lagged the eyebrows. At 270° of phase the mouth led the eyebrows. Both adapting and test faces were centred on a visual eccentricity of 3.125° of visual angle (DVA) and subtended 5 DVA. The test phases were randomised over trials. The adapt phases were presented in separate randomised blocks. Participants also completed a no adaptation control condition. All faces were presented upright. Participants completed a total of 800 trials over five blocks, providing 40 trials per adapting condition per test face relative timing.

An eye tracker was employed to enforce fixation during each trial’s test stage. A drift correction, requiring participants to fixate for 600 ms, was calculated prior to presentation in each trial. Gaze had to remain within a small central rectangle during stimulus presentation. Should fixation lapse, the request for a response would be replaced by an “Eye movement detected” message and the trial was appended to the end of the block for repetition. Otherwise, each participant response was followed by feedback (correct/incorrect) and a counter of completed/total trials.

#### Results and discussion

**Figure 2:**
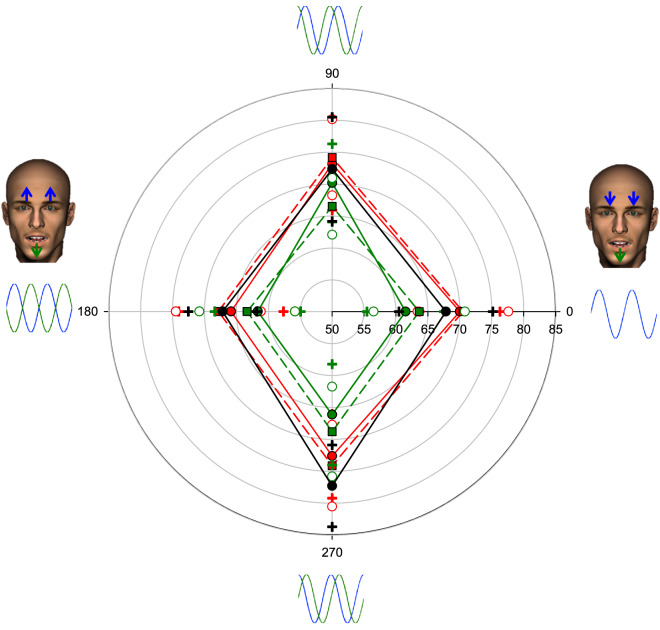
Percent correct for eyebrow wave detection is plotted along the radial axis (starting at 50% correct) as a function of relative phase at test. Eyebrows and mouths oscillated vertically with sinusoidal movement profiles. The diamond shape reflects the poorer detection performance for synchronous test faces as compared to asynchronous test faces (*F*(3, 42) = 15.76, *p* < .001, η_p_^2^ = 0.53). Adaptation to asynchronous feature motion (green) reduces discriminability of misaligned eyebrows across all test relative feature timings indicated by a main effect of adaptation type (*F*(4, 56) = 6.90, *p* < .001, η_p_^2^ = 0.33) and a non-significant interaction (*F*(12, 168) = 0.81, *p* = .645, η_p_^2^ = 0.05). Adaptation to synchronous feature motion (red) does not alter performance relative to the no adaptation control (black). Continuous red = 0°, dashed red = 180°, Continuous green = 90°, dashed green = 270° adaptation conditions. Crosses (continuous lines) and open circles (dashed lines) in matching colours show upper and lower 95% confidence limits. The stimuli were generated using Poser Pro 9 (SmithMicro; https://www.posersoftware.com).

The data in Fig. 2 show the percent correct for detecting the eyebrow wave. First, irrespective of the type of adaptation, and for the no adaptation control condition, performance depended upon test phase (*F*(3, 42) = 15.76, *p* < 0.001, η_p_^2^ = 0.53). Detection was impaired when the mouth and eyebrow motion was synchronous as compared to asynchronous, as indicated by the unequal diagonals of the diamond in Fig. 2. This demonstrates participants found it more difficult to identify the eyebrow wave in the presence of synchronous holistic motion. Second, there was a significant main effect of adaptation condition (*F*(4, 56) = 6.90, *p* < 0.001, η_p_^2^ = 0.33) with asynchronous adaptors reducing eyebrow wave detection to the same degree across all test conditions (non-significant interaction, *F*(12, 168) = 0.81, *p* = 0.645, η_p_^2^ = 0.05), shrinking the diamond. Detection performance after synchronous adaptation did not differ from the no adaptation control condition. Note that in the adaptation phase, the movement of the eyebrows was identical across synchronous and asynchronous adaptation regimes. Thus, the adaptation effects described here are phase-dependent. We found no evidence of phase-specific dynamic expression channels, rather reduced performance appears to be limited to both asynchronous adaptation conditions. Thus, changes in direction need to be temporally misaligned for adaptation to occur but the sign of the change is not critical.

In Experiment 1 faces were presented upright. In order to check whether the upright configuration is necessary for the key results we performed a partial replication for both upright and inverted faces.

## Experiment 2

### Method

#### Participants

Sixteen healthy adults (8 males) took part in Experiment 2. This was chronologically the third experiment conducted. We collected participant data until we were unable to test further participants due to compliance with COVID-19 pandemic directives. We then analysed the existing data set. The results of two participants were discarded; one due to performing at ceiling and the other due to deviating from fixation on more trials for one condition than our exclusion criterion allowed (see procedure). The findings from the remaining 14 participants (6 males) are reported here. All participants provided written, informed consent. The study was approved by the Ethics Committees of the University of Nottingham. All procedures adhered to the guidelines of the Declaration of Helsinki, 2008. Participants were screened for corrected visual acuity (20/20 or above) using a Snellen Chart before participating.

#### Design and procedure

The design was the same as for Experiment 1 except for the changes detailed here. The adaptation stage used only 0° and 90° mouth-eyebrow phase offsets, however for each offset the block could now be presented either upright or inverted, giving 4 conditions (0°/90° × upright/inverted). In the inverted blocks both adaptor and test faces were inverted. The test faces again had mouth-eyebrow phase offsets of 0°, 90°, 180° and 270°. The phase misalignment of the eyebrows was increased to 18.5° to allow for the expected enhanced difficulty of processing inverted faces. For the analysis, the trials with test faces of 0° and 180° mouth-eyebrow offset were collapsed into a ‘synchronous’ condition, and the 90° and 270° offsets into an ‘asynchronous’ condition.

The trials were typically presented in blocks of 48 and participants typically completed one block of each condition in one session and then completed a second block of each condition in the reversed order in a second session on a different day. Each session lasted 1 h. There was some variation in the number of trials completed within a block and the number of runs completed for some conditions, largely due to failures in eye tracking. However, all participants completed at least 48 trials in each of the four conditions. If a participant completed less than 48 trials for any condition, then all of their data was discarded.

Eye-tracking was again used to monitor fixation, and any blocks where there were 12 or more lapsed trials (25% of the number of trials) were discarded.

#### Results and discussion

**Figure 3:**
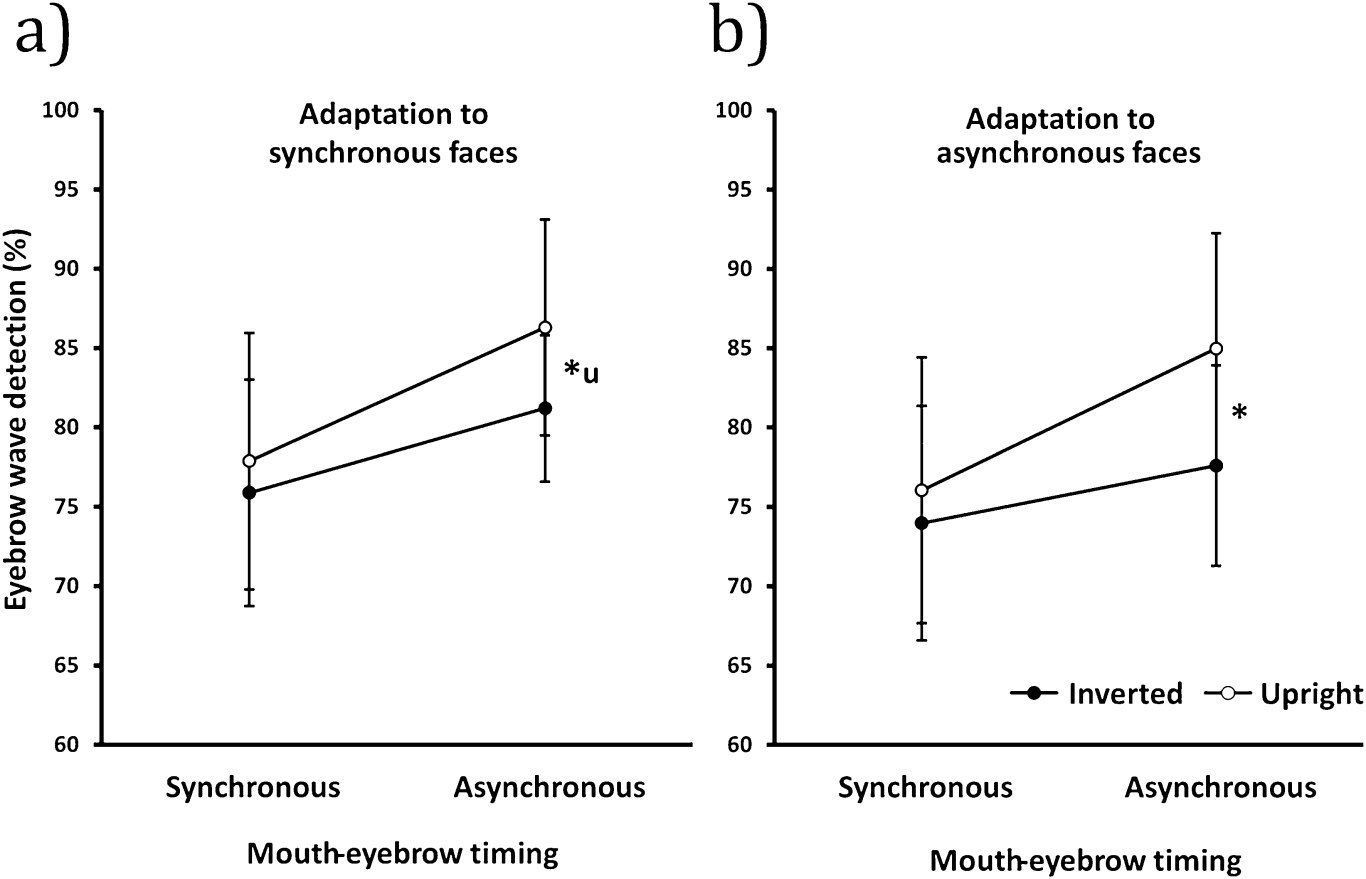
Percent correct for eyebrow wave detection as a function of test synchronicity after **(a)** adaptation to synchronous facial motion, and **(b)** adaptation to asynchronous facial motion. Closed circles = inverted faces, open circles = upright faces. Overall the eyebrow wave was harder to detect during synchronous test movement as in Experiment 1 (*F*(1,13) = 16.27, *p* = .001, η_p_^2^ = 0.56). Also, performance was reduced after asynchronous adaptation relative to synchronous adaptation (*F*(1,13) = 4.93, *p* = .045, η_p_^2^ = 0.28). There was also a main effect of orientation indicating an overall face inversion effect, with impaired eyebrow wave detection for inverted faces (*F*(1,13) = 5.93, *p* = .030, η_p_^2^ = 0.31). However, the inversion effect was greater for asynchronous tests than synchronous tests (*F*(1,13) = 6.90, *p* = .021, η_p_^2^ = 0.35) and it did not depend upon adaptation conditions (*F*(1,13) = .248, *p* = .627, η_p_^2^ = 0.02). Post-hoc paired-samples t-tests indicated that the inversion effect for asynchronous test faces when adapted to synchronous faces tended towards significance (*t*(13) = 2.17, *p* = .049, *d* = 0.58) but not did not survive correction for multiple comparisons (Bonferroni-corrected alpha = 0.0125). There was however a significant inversion effect for asynchronous test faces after adaptation to asynchronous faces (*t*(13) = 3.61, *p* = .003, *d* = 0.96). Error bars show 95% confidence intervals. * = *p* < .05. u = significant when uncorrected.

The data are shown in Fig. 3. Eyebrow wave detection performance was measured for synchronous and asynchronous test facial motion patterns after adaptation to synchronous and asynchronous faces. We performed a 2 × 2 × 2 repeated-measures ANOVA with orientation (upright versus inverted), adaptation phase (synchronous versus asynchronous) and test phase (synchronous versus asynchronous) as independent variables. We found that the eyebrow wave was harder to detect during synchronous test movement as before (*F*(1,13) = 16.27, *p* = 0.001, η_p_^2^ = 0.56). In addition, performance was reduced after asynchronous adaptation relative to synchronous adaptation (*F*(1,13) = 4.93, *p* = 0.045, η_p_^2^ = 0.28), replicating Experiment 1. There was also a main effect of orientation indicating an overall face inversion effect, with impaired eyebrow wave detection for inverted faces (*F*(1,13) = 5.93, *p* = 0.030, η_p_^2^ = 0.31). The presence of an overall inversion effect supports the view that the manipulations target face-specific mechanisms. Neither the interaction between adaptation and test synchrony nor the three-way interaction were significant. However, the inversion effect was greater for asynchronous tests than synchronous tests (interaction: *F*(1,13) = 6.90, *p* = 0.021, η_p_^2^ = 0.35) and it did not depend upon adaptation conditions (interaction: *F*(1,13) = 0.248, *p* = 0.627, η_p_^2^ = 0.02).

Post-hoc paired-samples t-tests further elaborated on the interaction between orientation and test synchrony, revealing that there was no significant inversion effect for synchronous test faces when adapted to either synchronous (*t*(13) = 0.76, *p* = 0.462, *d* = 0.20) or asynchronous (*t*(13) = 0.74, *p* = 0.470, *d* = 0.20) faces. The inversion effect for asynchronous test faces when adapted to synchronous faces tended towards significance (*t*(13) = 2.17, *p* = 0.049, *d* = 0.58) but not did not survive correction for the four multiple comparisons (Bonferroni-corrected alpha = 0.0125). There was however a significant inversion effect for asynchronous test faces after adaptation to asynchronous faces (*t*(13) = 3.61, *p* = 0.003, *d* = 0.96). Better performance for asynchronous tests overall is consistent with the view that asynchronous motion breaks down holistic encoding allowing the eyebrow wave to be detected more easily. The inversion effect may be due to impaired eyebrow wave detection in inverted faces or enhanced adaptation for inverted faces due to greater feature dissociation. The lack of an inversion effect for synchronous tests suggests synchrony groups features in both upright and inverted faces. The inversion effect for asynchronous tests after asynchronous adaptation suggests asynchrony combines with face inversion to provide greater feature dissociation and consequently greater feature-based adaptation.

Taken together Experiments 1 and 2 provide no support for a phase specific population code for relative feature motion. However, although the data distinguish synchronous and asynchronous motion processing, the lack of an effect of synchronous adaptation on eyebrow wave detection and more specifically the lack of a synchrony-specific adaptation effect does not provide sufficient evidence for conjoint coding of phase by synchronous and asynchronous systems either. However, we can conclude that adaptation to a particular facial motion will not degrade access to all aspects of facial motion for that stimulus, rather, adaptation to eyebrow movement, which is constant across conditions, is modified by the relative motion of other parts of the face, in this case the mouth and jaw. Adaptation to dynamic features (eyebrow position oscillation), which extends to an unadapted motion pattern of these features (eyebrow wave), is modified by dynamic facial grouping processes.

Simultaneous presentation of targets and foils requires attention to be divided between the two sides of the visual field. The task can also be performed by choosing a side and deciding whether the target is present or absent. To control for any effects of divided attention, and to limit the cognitive strategies that participants might adopt, we repeated the control (no adaptation) experiment, but this time simplifying the design by presenting the test faces to only one visual field. This required us to adopt a two-interval forced choice task. We also had the impression that the task might be easier on one side or another. This would suggest that there may be visual field differences in processing dynamic faces. Divided field studies have been used to probe laterality behaviourally^22^ and the two-interval forced choice paradigm allowed us to compare performance in the left and right visual fields. To anticipate the result, we found a reduction in eyebrow-wave detection performance for synchronous stimuli in the right visual field but no effect for the left visual field.

## Experiment 3

### Method

#### Participants

Twenty-two healthy adults (7 male) participated in the experiment. The data were collected in part to fulfil a final year project requirement and data collection was limited by deadlines for project submission. All participants provided written, informed consent. The study was approved by the Ethics Committees of the University of Nottingham. All procedures adhered to the guidelines of the Declaration of Helsinki, 2008. Participants were screened for corrected visual acuity (20/20 or above) using a Snellen Chart before participating.

#### Design and procedure

Participants performed a two-interval forced choice discrimination task (Fig. 1b). This task was identical to the control condition of Experiment 1 except the two test faces were presented to the same side of the visual field either to the left or right of fixation. The location of the faces alternated over trials. Again, the eyebrows and mouths oscillated sinusoidally at 1.5 Hz, raising and lowering in the case of the eyebrows and opening and closing, in the case of the mouths. The participants’ task was to report, by a keypress, which interval contained a face with a temporal misalignment of 12.5° of phase in its eyebrows. There were four global phase conditions randomly interleaved. The mouths were offset relative to the eyebrows by 0°, 90°, 180° or 270°. Both faces were presented either upright or inverted in randomised blocks. Participants completed a total of 640 trials over 20 blocks, providing 40 trials per feature relative timing per visual field per orientation. Eye tracking was again employed to enforce fixation during test face presentation.

#### Analysis

An initial 4 × 2 × 2 repeated-measures ANOVA with feature relative timing, visual field and orientation as independent variables yielded a significant main effect of feature relative timing (*F*(3, 63) = 4.05, *p* = 0.011, η_p_^2^ = 0.16) and a significant timing × visual field interaction (*F*(3, 63) = 4.32, *p* = 0.008, η_p_^2^ = 0.17). Neither the main effect of orientation nor its interactions were significant (orientation: *F*(1, 21) = 2.58, *p* = 0.123, η_p_^2^ = 0.11; orientation × visual field: *F*(1, 21) = 0.13, *p* = 0.73, η_p_^2^ = 0.01; orientation × relative timing: *F*(3, 63) = 0.31, *p* = 0.821, η_p_^2^ = 0.01; orientation × relative timing × visual field: *F*(3, 63) = 0.70, *p* = 0.555, η_p_^2^ = 0.03). We therefore collapsed over orientation by taking the mean of each participant’s percent correct for upright and inverted trials within each relative timing and visual field condition. Outcomes of the ANOVA on this collapsed data set are reported in the Results and Discussion section. We then further collapsed over synchronous (0° and 180°) and asynchronous (90° and 270°) relative timings in the same manner to assess the differential effect of synchronicity in the two visual fields. The outcome of the ANOVA on this collapsed data (with synchronicity replacing relative timing) are reported below.

#### Results and discussion

**Figure 4:**
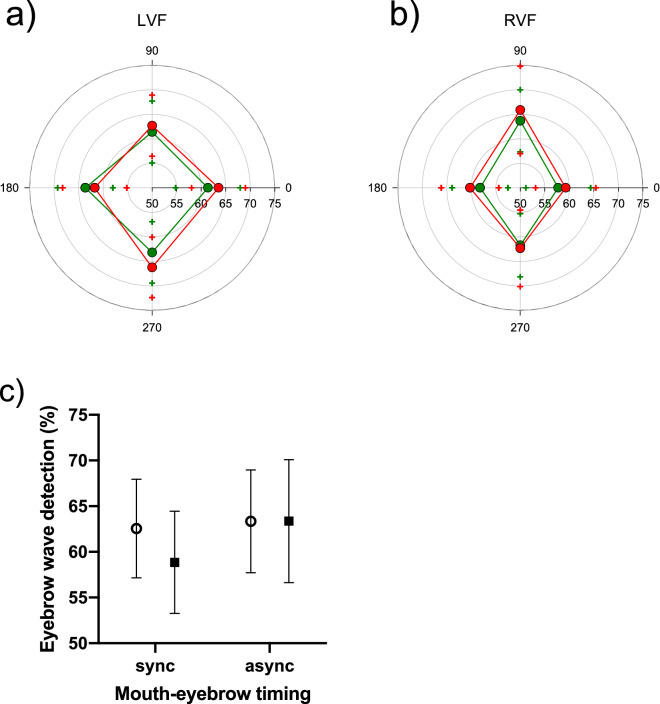
Percent correct for eyebrow wave detection is plotted along the radial axis (starting at 50% correct) as a function of relative phase at test. **(a)** Sensitivity to misaligned eyebrow movement did not differ significantly across face orientations (red = upright, green = inverted) or eyebrow-mouth relative timings in the left visual field. **(b)** Performance was poorer for synchronous eyebrow-mouth relative timings (0° and 180°) in the right visual field (*F*(3, 63) = 4.05, *p* = .01, η_p_^2^ = 0.16) supported by a significant hemifield × timing interaction, (*F*(3, 63) = 4.32, *p* = .01, η_p_^2^ = 0.17). Crosses in matching colours show upper and lower 95% confidence limits. **(c)** Data from **(a)** and **(b)** collapsed over orientations and feature synchronicity. Open circles = left visual field, solid squares = right visual field; sync = 0° and 180° collapsed, async = 90° and 270° collapsed. Eyebrow wave discriminability was selectively reduced by global facial synchrony in the right visual field (*F*(1, 21) = 9.41, *p* = .01, η_p_^2^ = 0.31) but not the left visual field, providing a significant hemifield × synchronicity interaction, (*F*(1, 21) = 5.24, *p* = .03, η_p_^2^ = 0.20). All error bars are 95% confidence intervals.

Participants had to indicate whether the first or second interval contained the eyebrow wave. The results are shown in Fig. 4. Eyebrow wave detection performance did not differ across the four feature timing relationships when presented in the left visual field (Fig. 4a). In the right visual field, however, performance was lower when mouth movement matched or opposed eyebrow movement (*F*(3, 63) = 4.05, *p* = 0.01, η_p_^2^ = 0.16) supported by a significant hemifield × timing interaction, (*F*(3, 63) = 4.32, *p* = 0.01, η_p_^2^ = 0.17). Figure 4c shows the data collapsed over the two types of synchronous and asynchronous motion. Eyebrow wave discriminability was selectively reduced by global facial synchrony in the right visual field (Fig. 4c; *F*(1, 21) = 9.41, *p* = 0.01, η_p_^2^ = 0.31) but not the left visual field, providing a significant hemifield × synchronicity interaction, (*F*(1, 21) = 5.24, *p* = 0.03, η_p_^2^ = 0.20).

There is some indication that dynamic facial expressions and facial speech may be differentially lateralised. A recent functional imaging experiment^23^ reported a greater BOLD response in the right posterior superior temporal sulcus (pSTS) for dynamic faces (chewing, fear) as compared to scrambled faces and a greater BOLD response in the left pSTS for visual speech as compared to chewing and fear expressions. In addition, Venezia, et al.^24^ found an area in the left posterior middle temporal gyrus that responded preferentially to both passive perception and rehearsal of speech cued by visual or audio-visual stimuli. The right visual field specialisation for synchronicity-mediated feature grouping found here indicates that grouping of dynamic features by synchronous motion may be particularly significant for facial speech processing.

## General discussion

To summarise the experimental results, we found that it was easier to detect an eyebrow wave when the eyebrows and mouth moved asynchronously rather than synchronously in all three experiments. In Experiment 3 this result was limited to stimuli presented to the right visual field.

Detection was reduced after adaptation to asynchronous adaptation as compared to synchronous adaptation in both Experiments 1 and 2. In Experiment 1 we found no difference between synchronous adaptation and the no adaptation control condition.

We compared detection for upright and inverted faces in Experiments 2 and 3. In Experiment 3, where we had no adaptation, we did not find an inversion effect for eyebrow wave detection. This suggest the inversion effect in Experiment 2 is driven by differences in adaptation. In Experiment 2, there was no inversion effect for synchronous tests, irrespective of the type of adaptation. There was however evidence for an inversion effect for asynchronous tests, with impaired eyebrow wave detection in inverted faces particularly after asynchronous adaptation.

The reduced sensitivity to the eyebrow wave during synchronous facial motion, which was evident in all three experiments, is consistent with reduced access to features when features are grouped by higher order structure. For example static curved lines are more difficult to detect in a visual search task when these features are presented in a schematic facial configuration, in which they depict mouths and eyebrows, as compared to when they are randomly located^25^ and vernier acuity is impaired when lines are grouped with flanking lines^26^.The poorer detection of feature motion (the eyebrow wave) in the case of synchronous motion indicates that eyebrows and mouth movements are bound holistically to a greater degree in the case of synchronous motion of the mouth and eyebrows relative to asynchronous motion.

The lack of, or reduced, effect of adaptation for synchronous motion on the detection of the eyebrow wave indicates that the holistic motion of the face protects component features from adaptation. This type of effect has been reported for low-level perceptual grouping, in which adaptation is reduced when the adapted feature forms part of a group. For example, He et al.^27^ showed that the tilt aftereffect was reduced when the adaptor formed part of an amodally complete occluded diamond that appeared to move as a unit, as compared to a very similar arrangement in which the components of the diamond appeared to move independently. We should point out that, since the face moves non-rigidly, dynamic facial feature binding would require a more elaborate grouping principle.

Adaptation to synchronous facial motion had no measurable effect in Experiment 1. The effect of adaptation to asynchronous facial motion reduced eyebrow wave detection in a way that was independent of the phase of the test pattern in both Experiments 1 and 2. The difference in the degree of adaptation induced by synchronous and asynchronous movements of the mouth and eyebrows on eyebrow wave detection indicates the pattern of global motion can modify the susceptibility of dynamic features to adaptation. Synchronous motion appears to support the binding of eyebrow and mouth movement. Adaptation to asynchronous motion appears to release the eyebrow motion from binding allowing adaptation of eyebrow motion as a separate feature.

It is generally agreed that upside-down faces are processed differently to upright faces. The main claims are that, for upright faces, spatial relations between features are encoded, typically referred to as configural coding, or that features are subsumed within a more global face representation, referred to as holistic coding, whereas pictorially rotated faces are encoded as spatially localised individuated features^28^. The idea that distances between features form the basis of a structural code does not bare close scrutiny^29,30^. The two critical observations supporting holistic coding for static faces are the composite effect^31^ and the part-whole effect^32^. In both cases grouping of face parts in the upright face is diminished by inverting the face allowing better part identification. Note, inversion does not tend to reduce recognition for features in isolation^33^.The question addressed here is, how do local features become bound together into a global representation in the upright face. The likely principle is that aspects of the face that change together group and this might apply across time scales from dynamic facial expressions to aging.

In low-level motion processing, spatially distributed features can be grouped if the local motion is similar, or if there is a plausible single global rigid motion^34,35^. Both image motion and object motion could lead to grouping at their different levels of representation. In the case of faces, grouping should be at the level of object motion, which describes change in a model of how faces vary. At this level, the coordinated non-rigid motion of the face provides a basis for grouping local features together^8,36^. In Experiment 3, which did not include an adaptation stage, we found no effect of inversion on eyebrow wave detection suggesting that the synchrony-based grouping of facial features, making component features harder to detect, occurs dynamically for both upright and inverted faces. In Experiment 2 we found a main effect of adaptation, indicating that synchronous motion protects embedded features from adaptation in both upright and inverted face. These results imply that grouping by synchrony is not specific to upright faces. It therefore appears to support dynamic binding in both upright and inverted faces. However, we also found the effect of inversion was greater for asynchronous tests particularly after asynchronous adaptation. We attribute this to a greater isolation of features when face inversion and feature asynchrony combine, as in the case of inverted asynchronous tests after adaptation to asynchronous motion, leading to stronger feature-based adaptation.

In Experiment 3 we found the synchrony impairment at test effect to be specific to stimuli presented to the right visual field. There is typically a left field advantage for face recognition^37,38^. Lateralisation of the synchrony effect to the right field, and by implication to the left hemisphere, suggests a link to facial speech perception^39,40^. The processing of facial expression can be disrupted by TMS delivered to left and right^41,42^ STS, and comparisons between moving faces and static faces tend to show activation in the right pSTS^23,43^. There is, however, recent evidence of greater activation in the left pSTS for speech-based expressions^23^ and an area in the left hemisphere designated the Temporal Visual Speech Area ventral and posterior to pSTS has similarly been identified as specialised for visual speech^40,44,45^. Masking by synchrony implies grouping of dynamic facial features. It is intriguing that both congenially deaf and hearing signers show lower motion coherence thresholds in the right visual field^46,47^. This low-level but global motion benefit suggest the sustained effort after recovering visual speech and signing information supports perceptual performance in the motion coherence task. There is also evidence of a right visual field benefit in object tracking^48^.

The Gestalt School’s principle of generalised common fate^49^ can be characterised as features that change together bind together. The corollary to that is that features that change asynchronously should separate. We have shown that for dynamic facial features (specifically, eyebrows and mouths), binding requires changes in direction to be temporally aligned but the sign of the change is not critical. The adaptation effects described here are relational and non-local as the local eyebrow motion was always the same for synchronous and asynchronous adaptors. There is no evidence for phase specific channels. Also, the evidence for specialised asynchronous and synchronous channels is weak, given that only asynchronous motion gives rise to feature adaptation. Rather, the dissociation in the effects of adaptation from synchronous and asynchronous motion is consistent with the idea that synchronous motion binds features into coordinated dynamic units whereas asynchronous motion separates them.

In conclusion, dynamic facial features can be bound together by synchronous motion, making the properties of individual features less accessible. Asynchronous motion releases dynamic features allowing feature motion adaptation.

## Data Availability

The datasets generated during and/or analysed during the current study are available from the corresponding author on reasonable request.
